# Precisão e Prognóstico da Hipertrofia Ventricular Esquerda Eletrocardiográfica e Ecocardiográfica em Adultos Brasileiros - Estudo ELSA-Brasil

**DOI:** 10.36660/abc.20250789

**Published:** 2026-05-06

**Authors:** Marcelo M. Pinto-Filho, Lillian Guimarães de Faria, Murilo Foppa, Angela Barreto Santiago Santos, Sandhi Maria Barreto, Antonio Luiz Pinho Ribeiro

**Affiliations:** 1 Universidade Federal de Minas Gerais Belo Horizonte MG Brasil Universidade Federal de Minas Gerais, Belo Horizonte, MG – Brasil; 2 Hospital das Clinicas Universidade Federal de Minas Gerais Belo Horizonte MG Brasil Hospital das Clinicas da Universidade Federal de Minas Gerais, Belo Horizonte, MG – Brasil; 3 Hospital de Clinicas de Porto Alegre Porto Alegre RS Brasil Hospital de Clinicas de Porto Alegre, Porto Alegre, RS – Brasil

**Keywords:** Eletrocardiografia, Hipertrofia Ventricular Esquerda, Prognóstico, Estudos Longitudinais

## Abstract

**Fundamento:**

A hipertrofia ventricular esquerda (HVE), definida pelo aumento da massa ventricular esquerda, é um preditor independente de mortalidade cardiovascular. Evidências sugerem que a HVE identificada por eletrocardiograma (ECG) e ecocardiografia representa entidades distintas com implicações prognósticas potencialmente diferentes.

**Objetivos:**

Avaliar a precisão diagnóstica dos critérios eletrocardiográficos para HVE em comparação com o ecocardiograma transtorácico (ETT) e avaliar seu valor preditivo para desfechos cardiovasculares fatais.

**Métodos:**

Realizamos uma análise transversal de acurácia diagnóstica e um acompanhamento prospectivo no âmbito do Estudo Longitudinal de Saúde do Adulto. A HVE no ECG foi definida pelos critérios do Código de Minnesota (CM), e a HVE no ETT pelo aumento da massa ventricular esquerda calculada. O nível de significância foi definido em 0,05.

**Resultados:**

Entre 2.849 participantes (43,6% homens), com mediana de idade de 62 anos, 12,5% tinham HVE definida por ETT e 5,9% por critérios eletrocardiográficos. A HVE definida por ECG apresentou, segundo diferentes critérios, sensibilidade muito baixa (5–12%), mas alta especificidade (95–99%) em comparação com a ETT. Nas análises prognósticas, a HVE definida por ETT se associou a eventos cardiovasculares adversos fatais (FMACE) significativos (HR 3,2; IC 95% 1,7–5,9). A HVE definida por ECG também previu FMACE, com HR próximo a 3,5 considerando os diferentes critérios do CM na análise de regressão multivariada. A capacidade preditiva se manteve significativa mesmo após ajuste adicional para hipertrofia definida por qualquer um dos métodos.

**Conclusões:**

Embora os critérios eletrocardiográficos tenham demonstrado baixa acurácia diagnóstica para HVE em comparação com o ETT, ambas as modalidades foram associadas independentemente a desfechos cardiovasculares adversos. Esses achados sugerem que a HVE definida por ECG e a definida por ETT fornecem informações prognósticas complementares e podem refletir processos fisiopatológicos distintos.

## Graphical abstract

**Figura Central:**
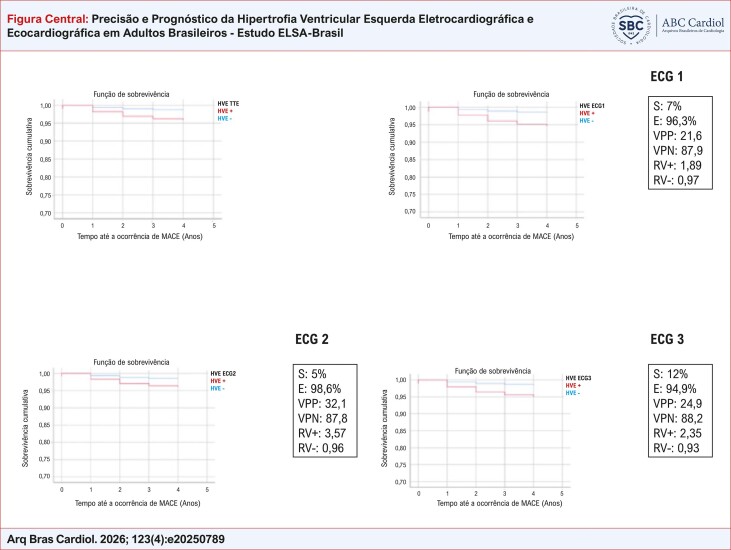
: Precisão e Prognóstico da Hipertrofia Ventricular Esquerda Eletrocardiográfica e Ecocardiográfica em Adultos Brasileiros - Estudo ELSA-Brasil Curvas de Kaplan-Meier para o tempo até o óbito devido à soma de infarto do miocárdio fatal e acidente vascular cerebral (FMACE) com métricas de concordância para cada critério eletrocardiográfico em comparação com o ecocardiograma. S: sensibilidade; E: especificidade; VPP: valor preditivo positivo; VPN: valor preditivo negativo; RV+: razão de verossimilhança positiva; RV: razão de verossimilhança negativa.

## O que já se sabe sobre este tema

Diversos parâmetros, incluindo ECG e exames de imagem cardíaca, podem diagnosticar a hipertrofia ventricular esquerda (HVE). Existe um importante debate sobre o significado clínico da hipertrofia elétrica versus a hipertrofia anatômica, e permanecem dúvidas se ambas fazem parte do mesmo processo patológico ou representam fenômenos clínicos distintos.

## O que este estudo acrescenta

Neste estudo com uma amostra de adultos brasileiros, demonstramos que há pouca sobreposição entre indivíduos com HVE definida por eletrocardiografia e por ecocardiografia, e que ambas estão relacionadas a desfechos cardíacos adversos, mesmo após ajuste mútuo em modelos de predição multivariados.

## Como este estudo pode afetar a pesquisa, a prática ou as políticas

Nosso estudo reforça a noção de que a hipertrofia elétrica pode ser uma entidade clínica distinta, em vez de simplesmente parte do processo de remodelação da hipertrofia anatômica. Esses achados ajudam os médicos a compreender vários aspectos do prognóstico de pacientes com hipertensão e demonstram a capacidade independente do ECG de fornecer um prognóstico mesmo na ausência de hipertrofia anatômica.

## Introdução

A sobrecarga hemodinâmica persistente é um dos principais determinantes da hipertrofia ventricular esquerda (HVE). Ela pode levar a adaptações anatômicas e elétricas inadequadas. A primeira pode ser avaliada pelo ecocardiograma transtorácico (ETT), que estima a massa e a geometria do ventrículo esquerdo (VE),^1^ o eletrocardiograma (ECG) mede a HVE por meio do registro da atividade elétrica. Apesar de serem frequentemente consideradas equivalentes, essas alterações podem ter origem em diferentes mecanismos adaptativos e resultar em manifestações distintas e, possivelmente, em diferentes consequências clínicas. Tradicionalmente, a HVE pode ser reconhecida no ECG de 12 derivações por alterações características no complexo QRS, segmento ST e ondas T. Numerosos critérios diagnósticos de HVE baseados nessas alterações foram desenvolvidos. A detecção de HVE pelo ECG pode ser importante para identificar lesão de órgãos-alvo na hipertensão e também por seu valor prognóstico na predição de futuras doenças cardiovasculares (DCV).^2,3^ Os diferentes critérios de HVE no ECG apresentam desempenho variável em relação à acurácia, especificidade e sensibilidade, com a maioria dos estudos relatando baixa sensibilidade e maior especificidade quando comparados ao ETT.^4^ Embora a baixa sensibilidade limite o valor do ECG como método de triagem para HVE na população geral (ou hipertensa), sua especificidade e possível informação prognóstica podem ser valiosas para identificar indivíduos com maior risco de DCV.^5^

Outro aspecto da HVE detectada por ECG é sua relação com a hipertrofia anatômica detectada por exames de imagem, como o ETT. O conceito de hipertrofia elétrica versus anatômica tem recebido crescente atenção e é objeto de significativo debate, pois pode nos informar sobre diferentes processos patológicos.^5^ Assim, os achados do ECG podem potencialmente fornecer informações independentes e talvez adicionais quando comparados a outras modalidades propedêuticas cardiovasculares, refletindo outras anomalias celulares subjacentes que vão além do diagnóstico anatômico de HVE. Como as DCV são a principal causa de morbidade e mortalidade em todo o mundo, esse exame onipresente pode desempenhar um papel essencial na assistência à saúde e no acesso a ela. O Estudo Longitudinal de Saúde do Adulto (ELSA-Brasil) é uma coorte ocupacional prospectiva que visa avaliar os determinantes de DCV e diabetes na população brasileira. O ECG e o ETT foram obtidos no início do estudo.

Nosso objetivo foi avaliar a HVE diagnosticada por ECG utilizando a definição do Código de Minnesota (CM) em relação aos seus parâmetros diagnósticos [ou seja, sensibilidade, especificidade, valores preditivos positivo e negativo (VPP e VPN), razões de verossimilhança positiva e negativa (RV+ e RV-)] quando comparada ao ETT nesta população. Além disso, buscamos avaliar o valor prognóstico de cada uma dessas duas ferramentas e se elas podem exercer essas capacidades preditivas independentemente uma da outra.

## Métodos

### Desenho do estudo e participantes

**Figura 1:**
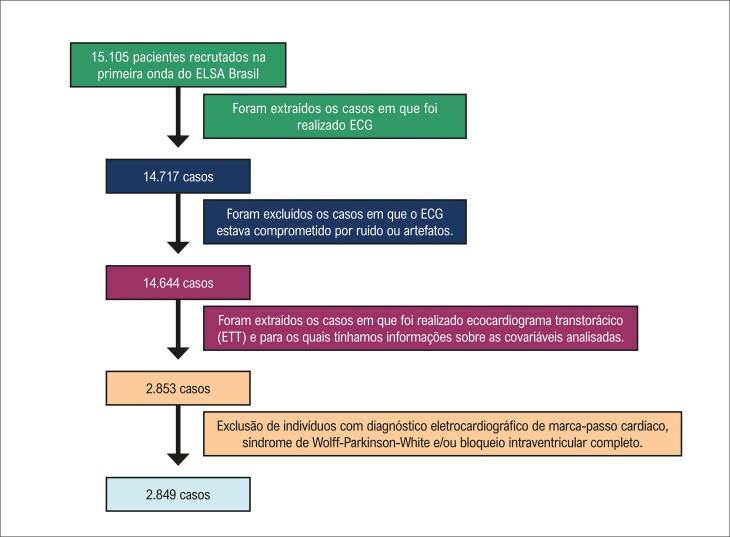
– Fluxograma de exclusão.

Este estudo foi concebido com duas abordagens complementares: um braço longitudinal prospectivo e um braço transversal de acurácia diagnóstica. A amostra do estudo foi derivada da linha de base do ELSA-Brasil, uma coorte multicêntrica recrutada entre 2008 e 2010, composta por 15.105 funcionários ativos e aposentados de seis universidades públicas e instituições de pesquisa localizadas nas regiões Nordeste, Sul e Sudeste do Brasil, com idades entre 35 e 74 anos. O delineamento do estudo e as características basais foram descritos anteriormente.^6^ Os participantes foram considerados elegíveis se tivessem tanto um ECG interpretável quanto um ETT. O ETT foi oferecido a todos os participantes, e sua aquisição e leitura foram realizadas priorizando, primeiramente, os indivíduos mais velhos (acima de 60 anos de idade) e também uma subamostra aleatória de 10% da população restante. Indivíduos com marca-passo cardíaco, síndrome de Wolff-Parkinson-White (WPW) ou bloqueio de condução intraventricular completo (QRS >120 ms) no ECG foram excluídos. O fluxograma de exclusões está representado na Figura 1. Entre os participantes da visita inicial do ELSA-Brasil que realizaram ETT e tinham dados disponíveis para avaliação de HVE por ecocardiografia e eletrocardiografia, 2.926 foram inicialmente selecionados para análise. Quatro indivíduos com marca-passo, WPW ou bloqueio de condução intraventricular completo (QRS >120 ms) no ECG foram excluídos, restando 2.922 casos. Outros 73 participantes foram excluídos devido a dados de ECG ausentes ou não interpretáveis (resultantes de ruído e artefatos). A amostra final do estudo foi composta por 2.849 participantes.

### Aquisição de ECG

Técnicos treinados e certificados obtiveram ECGs padrão de 12 derivações. Todos os participantes foram examinados em um ambiente confortável, com preparação da pele quando necessário, e os eletrodos foram posicionados em locais anatômicos padronizados.^7^ Os registros foram transmitidos e armazenados no centro de leitura de ECG da Universidade Federal de Minas Gerais, em Belo Horizonte, Brasil. Foi utilizado um dispositivo digital (Atria 6100, Burdick, Cardiac Science Corporation, Bothell, WA, EUA), que fornece medições automatizadas da frequência cardíaca, duração, amplitude e eixos das ondas P, QRS e T, bem como intervalos QT e QT corrigido (QTc) e dispersão do QT. Os ECGs foram transmitidos do Atria 6100 para um servidor dedicado no Centro de Leitura CL-ELSA/MG, onde foram arquivados para posterior análise utilizando o sistema de gerenciamento de ECG Pyramis (versão 6.2.b, Cardiac Science Corporation, Bothell, WA, EUA), permitindo edição e medições de intervalos batimento a batimento. Todos os ECGs foram submetidos à análise automatizada das amplitudes das ondas e codificados de acordo com o CM.^8^ Os traçados classificados como anormais por CM foram revisados manualmente. Dada a falta de superioridade inequívoca de qualquer critério isolado de ECG, este critério não foi utilizado.^9^ Adotamos os critérios definidos por CM, visto que todos os traçados foram codificados de acordo com eles. A HVE no ECG foi definida pela presença dos códigos CM 3.1, 3.3 e sua combinação.

### Aquisição de ETT

Ecocardiogramas foram realizados em 19% dos participantes do ELSA-Brasil. O ETT foi realizado em indivíduos com idade superior a 60 anos e em uma subamostra aleatória de 10% da coorte total. Todos os exames seguiram protocolos de pesquisa padronizados.^10^ As imagens foram adquiridas utilizando um sistema Toshiba Aplio XG com um transdutor setorial de 2,5 MHz e transferidas para o Sistema de Arquivamento e Comunicação de Imagens (PACS) do ELSA. As análises foram realizadas no centro de leitura de ecocardiografia do ELSA-Brasil. As leituras incluíram avaliação qualitativa dos achados ecocardiográficos e medidas quantitativas da função sistólica e diastólica do VE, geometria e dimensões do VE, tamanho do átrio esquerdo, anormalidades regionais da motilidade da parede e valvopatias. A massa do VE foi calculada utilizando a fórmula de Devereux: $\mathrm{MVE}(g)=0,8\times [1,04\times (SIV+DIVd+\mathrm{PWTd}{)}^{3}-(\mathrm{DIVd}{)}^{3}]+0,6g$ , onde SIV é a espessura do septo interventricular, DIVd é o diâmetro interno do VE na diástole e PWTd é a espessura da parede posterior na diástole. Neste estudo, a HVE foi definida como índice de massa do VE >115 g/m^2^ em homens e >95 g/m^2^ em mulheres.

### Variáveis preditivas

Para avaliação da acurácia diagnóstica, as variáveis preditivas foram categorizadas como HVE por ECG. A HVE no ETT foi representada pela variável HVE_ETT e considerada o padrão ouro para o diagnóstico de HVE. Organizamos a HVE no ECG em três variáveis, duas das quais representavam um único critério de Minnesota: HVE_ECG1 (CM 3.1) e HVE_ECG2 (CM 3.3), e a terceira, a soma das duas, HVE_ECG3 (combinação de 3.1 + 3.3).

Com relação à análise prognóstica, para investigar o potencial valor preditivo incremental da combinação de informações de ECG e ETT, criamos uma variável composta HVE_ECG+ETT, definida como a soma de HVE_ETT e HVE_ECG3. Nesta análise, todos os outros preditores foram utilizados: HVE_ETT, HVE_ECG1, HVE_ECG2, HVE_ECG3 e HVE_ECG+ETT.

### Covariáveis

Nenhuma covariável foi definida para as análises de acurácia diagnóstica. Para os modelos prognósticos, as seguintes covariáveis foram incluídas: sexo (masculino, feminino); escolaridade (com ou sem ensino superior completo); faixa etária (35–44, 45–54, 55–64, 65–74 anos); hipertensão (pressão arterial sistólica (PAS) ≥140 mmHg e/ou pressão arterial diastólica (PAD) ≥90 mmHg, autodiagnóstico ou uso de medicação anti-hipertensiva); PAS; PAD; diabetes mellitus (diagnóstico definido, uso de medicamentos para o tratamento de diabetes, HbA1c >6,5%, glicemia de jejum >126 mg/dL ou glicemia pós-prandial >200 mg/dL); Dislipidemia, definida nesta análise como qualquer um dos seguintes: colesterol total >240 mg/dL, LDL >160 mg/dL, HDL <40 mg/dL ou uso de terapia hipolipemiante; tabagismo (≥100 cigarros ao longo da vida e tabagismo atual); e doença cardiovascular autorrelatada (insuficiência cardíaca, doença coronariana ou acidente vascular cerebral (AVC)).

## Resultados

Para as análises de acurácia diagnóstica, as variáveis de desfecho incluíram sensibilidade, especificidade, VPP, VPN, RV+ e RV- em comparação com a detecção de HVE pelo ETT. Para as análises prognósticas, utilizamos apenas os desfechos fatais, que eram os desfechos disponíveis no momento do nosso estudo (dezembro de 2013). Eles foram categorizados como infarto agudo do miocárdio (IAM) fatal, AVC fatal e sua soma (FMACE). Os desfechos foram avaliados pelos investigadores do ELSA-Brasil de acordo com critérios internacionalmente validados. O acompanhamento foi realizado por meio de entrevistas telefônicas anuais, complementadas, quando necessário, por consultas clínicas ou vinculação a bancos de dados nacionais de saúde, incluindo o Sistema de Informações sobre Mortalidade (SIM) e o Sistema de Informações Hospitalares (SIH) do Ministério da Saúde do Brasil.

### Análise estatística

Sensibilidade, especificidade, VPP, VPN, RV+ e RV- foram calculados para cada critério. Para as análises prognósticas, foram avaliadas as associações basais entre HVE no ECG e HVE no ETT. As curvas de sobrevida de Kaplan-Meier e o teste de log-rank foram utilizados para comparar a incidência cumulativa dos desfechos definidos entre os participantes com HVE definida por ECG e por ETT. Um modelo de riscos proporcionais de Cox foi aplicado para estimar as *Hazard ratios* (HRs) e os intervalos de confiança (ICs) de 95% para a associação da HVE por ECG e ETT separadamente com o desfecho primário. Em seguida, um modelo de regressão de Cox hierárquico foi construído, ajustando-se sequencialmente para sexo e idade (modelo 1), hipertensão, diabetes, tabagismo, dislipidemia (modelo 2) e, finalmente, doença cardiovascular autorrelatada (modelo 3). Como análise de sensibilidade adicional, foi realizada regressão de Cox com ajuste mútuo: a HVE ecocardiográfica foi incluída como covariável nos modelos em que ECG-HVE era o preditor, e vice-versa (modelo 4). As características da amostra foram resumidas utilizando estatísticas descritivas. Os dados clínicos foram inseridos em um banco de dados utilizando o pacote estatístico SPSS (*Statistical Package for the Social Sciences*), versão 22.0. As variáveis contínuas foram expressas como medianas e intervalos interquartis (Q1–Q3) e comparadas utilizando o teste de Mann-Whitney. As variáveis categóricas são descritas em termos de frequências e comparadas utilizando o teste do qui-quadrado. Um valor de alfa bicaudal <0,05 foi considerado estatisticamente significativo.

## Resultados

A população do estudo era composta majoritariamente por mulheres, com idade mediana de 62 anos e alto nível de escolaridade. Aproximadamente metade dos participantes apresentava hipertensão e a maioria, dislipidemia. A Tabela 1 resume as características clínicas e demográficas, e os principais achados estão resumidos na Figura Central.

**Tabela 1:** – Características clínicas e sociodemográficas da população do estudo

Características	População geral (2848)	HVE ETT	HVE ECG1	HVE ECG2	HVE ECG3	HVE ETT+ECG
HVE (%)	-	12,5	4,1	1,9	5,9	17
Participantes do sexo masculino (%)	46,3	41,7	58,6	66,0	60,9	46,9
Faixa etária (%)						
35 a 44 anos	7,1	1,1	6,9	17,0	10,1	4,1
45 a 54 anos	16,0	4,2	6,9	3,8	5,9	5,0
55 a 64 anos	43,1	44,0	41,4	39,6	40,8	44,0
65 - 74 anos	33,9	50,7	44,8	39,6	43,2	46,9
Idade (mediana)	62 (56 – 66)	65 (61 – 69)	63 (60 – 67,8)	64 (59 – 69)	63 (60 – 68)	64 (61 - 68)
Escolaridade						
Superior (%)	56	41,7	34,5	28,3	32,5	40,9
Hipertensão (%)	49,8	77,0	72,4	62,3	69,2	73,5
Pressão arterial sistólica (mediana)	123,5 (113 - 136)	135 (124 - 150)	135 (122 – 155)	135 (122 –153)	135 (122 - 154)	134 (122 - 150)
Pressão arterial diastólica (mediana)	75,5 (69 - 83)	78,5 (70 - 86)	79,3 (73- 88)	79,5 (71 - 88)	79,5 (72 - 88)	78,5 (71 - 87)
Tabagismo (%)	10,4	9,2	8,6	18,9	11,8	9,3
Diabetes (%)	26,2	43,3	37,1	24,5	33,1	40,4
Dislipidemia (%)	56,0	60,4	53,4	62,3	56,2	58,6
HDL baixo (%)	24,1	30,6	18,1	26,4	20,7	28,0
LDL alto (%)	36,0	39,9	31,9	39,6	34,3	37,5
DCV (%)	11,2	27,8	12,9	13,5	13,1	23,4

ETT: ecocardiograma transtorácico; HVE: hipertrofia ventricular esquerda; ECG: eletrocardiograma; PAS: pressão arterial sistólica; PAD: pressão arterial diastólica; HDL: lipoproteína de alta densidade; LDL: lipoproteína de baixa densidade; DCV: doenças cardiovasculares.

Dos 2.849 participantes, 12,5% apresentaram HVE à ecocardiografia. A HVE identificada pela definição HVE_ECG3 (soma de CM 3.1 + 3.3) foi a mais frequente, seguida por HVE_ECG1 e HVE_2. Essas frequências estão descritas na Tabela 2. A variável composta HVE_ECG+ETT (ecocardiografia transtorácica) identificou HVE em 17% dos participantes. Indivíduos com HVE, detectada por qualquer uma das modalidades, apresentaram maior probabilidade de ter hipertensão e dislipidemia. O desempenho de cada critério de HVE também está resumido na Tabela 2. Em geral, todos apresentaram baixa sensibilidade e alta especificidade, bem como menor VPP e maior VPN.

**Tabela 2:** – Desempenho do eletrocardiograma para o diagnóstico de hipertrofia ventricular esquerda em comparação com o ecocardiograma

	Anterior	S	E	VPP	VPN	RV+	RV -
ECG 1	4,1%	7%	96,3%	21,6	87,9	1,89	0,97
ECG 2	1,9%	5%	98,6%	32,1	87,8	3,57	0,96
ECG 3	5,9%	12%	94,9%	24,9	88,2	2,35	0,93

S: sensibilidade; E: especificidade; VPP: valor preditivo positivo; VPN: valor preditivo negativo; RV +: razão de verosimilhança positiva; RV-: razão de verosimilhança negative; ECG: eletrocardiograma.

**Figura 2:**
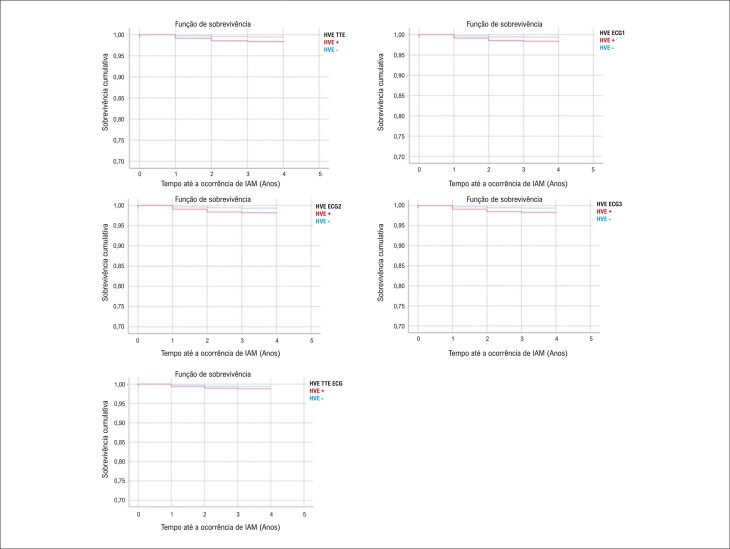
– Curvas de Kaplan-Meier para o tempo até a morte devido a infarto do miocárdio.

**Figura 3:**
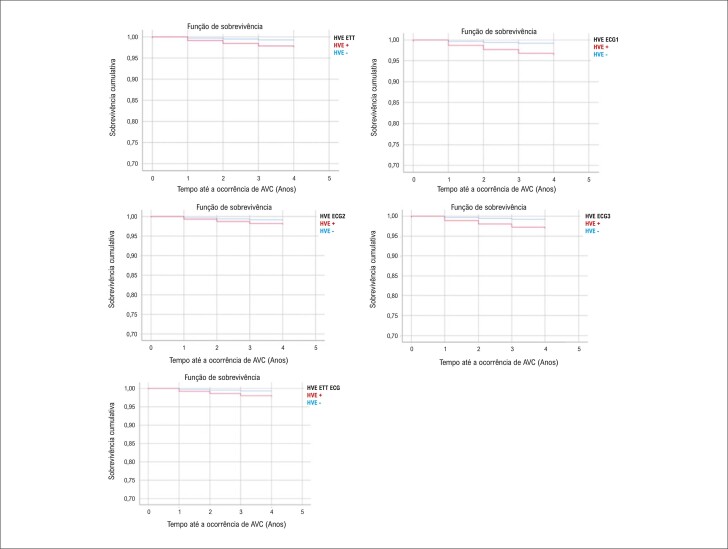
– Curvas de Kaplan-Meier para o tempo até o óbito por acidente vascular cerebral. AVC: acidente vascular cerebral.

Conforme demonstrado na Tabela 3, o HVE_ECG1 foi associado às maiores taxas de mortalidade por IAM, AVC e FMACE. A Tabela 4 mostra os modelos preditivos multivariados ajustados para sexo, idade, fatores de risco cardiovascular e HVE. Para a mortalidade por IAM isoladamente, nenhuma das variáveis permaneceu estatisticamente significativa no modelo final. No entanto, tanto o HVE_ECG1 quanto o HVE_ECG3 foram associados a um risco quase três vezes maior de mortalidade por AVC. Para o desfecho composto de mortalidade por IAM e AVC (FMACE), todas as variáveis, exceto o HVE_ECG2, foram associadas a um risco aumentado. As curvas de sobrevida de Kaplan-Meier para IAM, AVC e eventos fatais combinados são mostradas nas Figuras 2, 3 e 4, respectivamente.

**Tabela 3:** – Frequências dos desfechos na coorte geral e estratificadas por variáveis preditoras

Taxa de mortalidade	Todos	HVE ETT	HVE ECG 1	HVE ECG 2	HVEECG 3	HVE ETT + ECG
Morte por IAM	0,7%	1,7%	1,7%	1,9%	1,8%	1,2%
Morte por AVC	0,9%	2,2%	3,4%	1,9%	3,0%	2,1%
Morte combinada por AVC e IAM	1,6%	3,9%	5,2%	3,8%	4,7%	3,3%

IAM: infarto agudo do miocárdio; HVE: hipertrofia ventricular esquerda; ETT: ecocardiograma transtorácico; ECG: eletrocardiograma.

**Tabela 4:** – Modelos preditivos multivariados para os desfechos de morte por infarto do miocárdio, acidente vascular cerebral e morte combinada

Hazard ratio (HR) IC 95%	HVE ETT	HVE ECG 1	HVE ECG 2	HVE ECG 3	HVE ETT +ECG
**HR IAM**					
Modelo 1 ajustado por sexo e idade.	3.1 (1.2 – 8)	2,6 (0,6 – 11,4)	2,8 (0,4 – 20,9)	2,8 (0,8 – 9,6)	2.1 (0,8 – 5,5)
Modelo 2 = modelo 1 + fatores de risco CV*	2,9 (1,1 – 7,7)	2.1 (0,5 – 9,2)	2.1 (0,3 – 16)	2,2 (0,6 – 7,6)	1,9 (0,7 – 4,9)
Modelo 3 = modelo 2 + DCV prevalente	2,6 (0,9 – 7,3)	2.1 (0,5 – 9)	2.1 (0,3 – 15,7)	2,2 (0,6 – 7,5)	1,7 (0,6 – 4,6)
Modelo 4 = modelo 3 + HVE por ECG ou ETT	2,5 (0,9 – 6,9)	1,9 (0,4 – 8,2)	1,7 (0,2 – 12,6)	1,9 (0,5 – 6,5)	
**HR AVC**					
Modelo 1 ajustado por sexo e idade.	3.1 (1.3 – 7)	4.2 (1.4 – 12)	2.1 (0,3 – 15,3)	3,7 (1,4 – 9,7)	2,9 (1,3 – 6,4)
Modelo 2 = modelo 1 + fatores de risco CV*	2,7 (1,2 – 6,3)	3,6 (1,2 – 10,4)	1,7 (0,2 – 12,9)	3.1 (1,2 – 8,3)	2,6 (1,2 – 5,7)
Modelo 3 = modelo 2 + DCV prevalente	1,9 (0,8 – 4,6)	3,4 (1,2 – 9,9)	1,8 (0,2 – 13,1)	3 (1,2 – 8,1)	2 (0,9 – 4,4)
Modelo 4 = modelo 3 + HVE por ECG ou ETT	1,8 (0,7 – 4,3)	3.3 (1.1 – 9.5)	1,5 (0,2 – 11,5)	2,8 (1,1 – 7,6)	
**HR IAM + AVC**					
Modelo 1 ajustado por sexo e idade.	3,2 (1,7 – 5,9)	3,6 (1,5 – 8,4)	2,4 (0,6 – 10,1)	3.4 (1,6 – 7,3)	2,7 (1,5 – 4,9)
Modelo 2 = modelo 1 + fatores de risco CV*	2,8 (1,5 – 5,4)	3 (1,3 – 7)	2 (0,5 – 8,1)	2,8 (1,3 – 6)	2,3 (1,3 – 4,3)
Modelo 3 = modelo 2 + DCV prevalente	2.3 (1.2 – 4.4)	2,9 (1,2 – 6,8)	1,9 (0,5 – 8,1)	2,7 (1,3 – 5,8)	1,9 (1,1 – 3,6)
Modelo 4 = modelo 3 + HVE por ECG ou ETT	2.1 (1.1 – 4.1)	2,7 (1, 1 – 6,4)	1,6 (0,4 – 6,7)	2.4 (1.1 – 5.3)	

IAM: infarto agudo do miocárdio; HVE: hipertrofia ventricular esquerda; ETT; ecocardiograma transtorácico; ECG: eletrocardiograma; CV: cardiovascular; DCV: doenças cardiovasculares. *Os fatores de risco para ajuste foram hipertensão, diabetes, tabagismo e dislipidemia.

**Figura 4:**
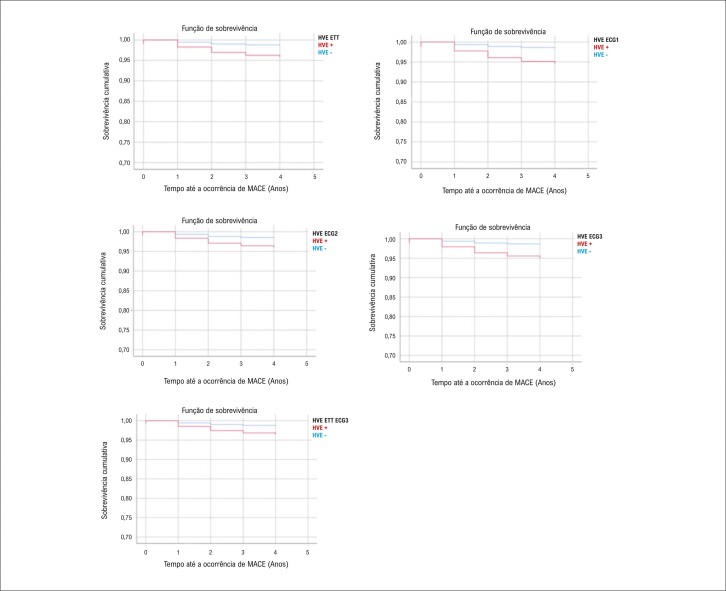
– Curvas de Kaplan-Meier para o tempo até a morte devido à soma de infarto do miocárdio fatal e acidente vascular cerebral (FMACE).

Nas análises de sensibilidade, ajustando os modelos ETT_HVE para ECG_HVE e vice-versa, a significância preditiva para mortalidade por AVC de HVE_ECG1 e HVE_ECG3, conforme observado no modelo 3, permaneceu significativa. Da mesma forma, no modelo 4, as variáveis previamente associadas a FMACE no modelo 3 mantiveram a significância estatística.

## Discussão

Os critérios eletrocardiográficos para HVE, quando comparados ao ETT como padrão ouro, demonstraram baixa acurácia diagnóstica, atribuída principalmente à baixíssima sensibilidade. Além disso, as razões de verossimilhança obtidas foram desfavoráveis, com valores positivos abaixo de cinco e valores negativos próximos a 1,0. Como as razões de verossimilhança integram sensibilidade e especificidade, são consideradas parâmetros superiores para avaliar a capacidade de um teste em influenciar corretamente o raciocínio clínico quanto à presença ou ausência da doença. A baixa concordância dos critérios eletrocardiográficos para HVE observada em nosso estudo é consistente com evidências anteriores, incluindo uma revisão sistemática de estudos de acurácia diagnóstica sobre critérios eletrocardiográficos para HVE.^4^ As sensibilidades medianas relatadas variaram de 10,5% para o índice de Gubner a 21% para o índice de Sokolow-Lyon, enquanto as especificidades medianas variaram de 89% (Sokolow-Lyon) a 99% (Romhilt-Estes, limiar de cinco pontos). Levy et al.,^9^ no *Framingham Heart Study*, avaliaram 4.684 participantes e relataram uma sensibilidade geral do ECG para HVE de 6,9% e uma especificidade de 98,8%. Da mesma forma, no estudo MESA, a avaliação de 13 critérios de HVE baseados em ECG revelou sensibilidade consistentemente baixa (10–26%) e alta especificidade (88–99%).^11^

Um método diagnóstico com sensibilidade tão baixa (apesar da alta especificidade) provavelmente não discriminará adequadamente entre pacientes afetados e indivíduos saudáveis, tendendo, em vez disso, a classificar a maioria como saudável, limitando, assim, sua utilidade clínica como ferramenta diagnóstica. Portanto, apesar de ser barato, amplamente disponível e isento de efeitos adversos diretos, o ECG não é um método eficaz de triagem para HVE. Pelo menos, quando se utilizam os critérios tradicionais de ECG para HVE e se toma a hipertrofia anatômica por ETT como padrão de referência.

Em relação às implicações prognósticas, embora a HVE definida por ECG tenha apresentado fraca correlação com a HVE ecocardiográfica, ela permaneceu significativamente associada a desfechos cardiovasculares fatais. Tanto o ETT quanto o ECG foram preditores robustos de morte por IAM ou AVC, mesmo após ajuste para fatores de risco cardiovascular convencionais e doença cardiovascular estabelecida. Nenhum dos métodos manteve significância estatística no modelo final para mortalidade por IAM, embora uma forte tendência tenha persistido para a HVE definida por ETT (HR 2,6, IC 95% 0,9–7,3). Esses achados podem ser parcialmente explicados pelo baixo número desses eventos e pela associação mais estreita da HVE com a hipertensão e, portanto, pelas consequências mais intimamente relacionadas à hipertensão, particularmente o AVC.

As análises de sensibilidade reforçaram o valor independente de cada modalidade: a HVE definida por ECG permaneceu um preditor significativo mesmo após ajuste adicional para HVE definida por ETT, e vice-versa. Além disso, a variável combinada (HVE por ECG e ETT) não apresentou associações mais fortes com desfechos adversos do que as modalidades individuais, sugerindo complementaridade em vez de valor preditivo aditivo. Em consonância com nossos achados, Sundström et al. demonstraram que a HVE definida por ECG foi um forte preditor de mortalidade por todas as causas, mesmo após ajuste para HVE ecocardiográfica.^12^ Da mesma forma, Leigh et al. relataram que a coexistência de HVE definida por ECG e ETT estava associada a um risco aumentado de eventos cardiovasculares.^13^ Ao mesmo tempo, Patel et al. descobriram que ambas as modalidades previam independentemente a incidência de fibrilação atrial, não obstante os fatores de risco estabelecidos.^14^ Notavelmente, a capacidade preditiva da HVE definida por ECG não dependeu da presença de HVE ecocardiográfica, corroborando seu papel como marcador eletrofisiológico de anormalidades cardíacas, independente da hipertrofia anatômica. Essa baixa concordância entre as duas modalidades, ao mesmo tempo que mantêm informações prognósticas semelhantes, indica que, talvez, ambas as entidades representem um sinal real de anormalidades cardiovasculares.

Em conjunto, esses achados corroboram a hipótese de que o ECG e o ETT podem captar aspectos distintos da patologia cardíaca, que poderiam ser descritos como sobrecarga elétrica (definida pelo ECG) versus adaptação anatômica à sobrecarga (definida pelo ETT). Vale ressaltar também que o ETT identifica o aumento da massa do VE e não a hipertrofia miocárdica em si, o que significa que outros processos de aumento de massa (como o que ocorre na amiloidose) também levam a um aumento da massa do VE. Outra possível explicação para essa discrepância é que a HVE definida pelo ECG é impulsionada principalmente por critérios de voltagem (em nosso estudo), que podem ser influenciados por fatores externos (como formato do tórax ou outras comorbidades). A presença de um sinal prognóstico semelhante ao da HVE definida pelo ETT aponta para um fenômeno cardíaco potencialmente intrínseco. Estudos anteriores demonstraram que as alterações do QRS na HVE definida pelo ECG representam uma combinação de remodelamento estrutural e elétrico.^15,16^ Em contraste, a HVE ecocardiográfica é quase inteiramente determinada pelo aumento da massa ventricular esquerda ou, ocasionalmente, como fator de confusão, pela representação de aumento de massa não relacionado à hipertrofia verdadeira, como na amiloidose. Nesse contexto, o ECG não deve ser visto meramente como um método menos sensível para detectar HVE. Se assim fosse, nenhum valor prognóstico adicional seria esperado quando a HVE definida por ECG é considerada juntamente com a HVE ecocardiográfica. Corroborando essa interpretação, nosso estudo mostrou pouca sobreposição entre os participantes que atendiam aos critérios de HVE por ECG versus ETT e nenhuma força preditiva incremental para doença cardiovascular fatal quando uma variável combinada foi testada. Assim, apesar das potenciais diferenças na patologia subjacente, tanto a HVE definida por ECG quanto a definida por ETT forneceram informações prognósticas igualmente relevantes em relação ao risco de AVC. Isso reforça o valor clínico do ECG, especialmente em locais com acesso limitado a diagnósticos cardiovasculares avançados ou especialistas em cardiologia. Estudos prospectivos futuros são necessários para esclarecer se a HVE definida por ECG reflete uma forma precoce de remodelação elétrica que precede as alterações anatômicas, ou se representa uma entidade patológica distinta.

Nossos resultados devem ser interpretados considerando algumas limitações. Primeiro, analisamos apenas desfechos fatais, resultando em um número relativamente baixo de eventos e amplos intervalos de confiança para os efeitos estimados. Segundo, definimos a HVE baseada em ECG utilizando o CM, que, embora amplamente aplicado em estudos populacionais há décadas, é menos relevante para a prática clínica de rotina. Critérios clínicos comumente utilizados, como os de Cornell, Sokolow-Lyon ou Romhilt-Estes, poderiam ter produzido resultados mais diretamente aplicáveis à prática. No entanto, o uso do CM foi pragmático, dada a sua aplicação consistente em nossa coorte. Da mesma forma, a HVE ecocardiográfica foi definida como massa ventricular esquerda indexada >115 g/m^2^ para homens e >95 g/m^2^ para mulheres; a análise dicotômica é menos sensível do que a contínua. Por fim, a estratégia de amostragem do ETT (10% de uma subamostra aleatória da coorte mais os participantes com idade >60 anos) pode ter introduzido viés de seleção, e nossa coorte, composta por servidores públicos de universidades e instituições de pesquisa, pode não ser totalmente representativa da população brasileira em geral. Os pontos fortes do nosso estudo incluem o uso de desfechos objetivos e concretos, minimizando assim o viés de mensuração, a padronização da aquisição de ECG e ETT e a aplicação de modelos multivariados ajustados para potenciais fatores de confusão, melhorando a acurácia das estimativas de efeito.

## Conclusão

A HVE definida por ECG apresentou menor acurácia diagnóstica na identificação da HVE detectada por ecocardiografia transtorácica (ETT); contudo, ambas as modalidades foram associadas independentemente a desfechos cardiovasculares adversos, potencialmente de forma complementar. Mais pesquisas são necessárias para esclarecer o valor prognóstico de diferentes marcadores de HVE e para melhor compreender a relação — e possível sobreposição — entre a hipertrofia elétrica e anatômica.
