# Supplementary material for: Precisão e Prognóstico da Hipertrofia Ventricular Esquerda Eletrocardiográfica e Ecocardiográfica em Adultos Brasileiros - Estudo ELSA-Brasil
Source: Arq Bras Cardiol. 2026 May 6;123(4):e20250789. [Article in Portuguese] doi: 10.36660/abc.20250789 (PMC13398829; doi:10.36660/abc.20250789)
Supplement: Translation

# Translation: Accuracy and Prognosis of Electrocardiographic and Echocardiographic Left Ventricular Hypertrophy in Brazilian Adults - ELSA-Brasil Study

**DOI:** 10.36660/abc.20250789i

**Authors:** Marcelo M. Pinto-Filho, Lillian Guimarães de Faria, Murilo Foppa, Angela Barreto Santiago Santos, Sandhi Maria Barreto, Antonio Luiz Pinho Ribeiro

**Affiliations:** 1 Brazil Universidade Federal de Minas Gerais, Belo Horizonte, MG – Brazil; 2 Brazil Hospital das Clinicas da Universidade Federal de Minas Gerais, Belo Horizonte, MG – Brazil; 3 Brazil Hospital de Clinicas de Porto Alegre, Porto Alegre, RS – Brazil

**Keywords:** Electrocardiography, Left Ventricular Hypertrophy, Prognosis, Longitudinal Studies

## Abstract

**Background:**

Left ventricular hypertrophy (LVH), defined by increased left ventricular mass, is an independent predictor of cardiovascular mortality. Evidence suggests that LVH identified by electrocardiogram (ECG) and echocardiography represents distinct entities with potentially different prognostic implications.

**Objectives:**

Assess the diagnostic accuracy of electrocardiographic criteria for LVH compared with transthoracic echocardiogram (TTE) and to evaluate their predictive value for fatal cardiovascular outcomes.

**Methods:**

We conducted a cross-sectional diagnostic accuracy analysis and a prospective follow-up within the Longitudinal Study of Adult Health. LVH on ECG was defined by Minnesota Code criteria, and LVH on TTE by increased calculated left ventricular mass. Significance level was defined at 0,05.

**Results:**

Among 2,849 participants (43.6% male), median age 62 years, 12.5% had LVH by TTE and 5.9% by ECG criteria. ECG-defined LVH showed, from different criteria, very low sensitivity (5–12%) but high specificity (95–99%) compared with TTE. In prognostic analyses, TTE-defined LVH was associated with significant fatal adverse cardiovascular events (FMACE) (HR 3.2, 95% CI 1.7–5.9). ECG-defined LVH also predicted FMACE, with HR close to 3.5 across the different MC criteria in multivariate regression analysis. The predictive capacity remained significant after further adjustment for hypertrophy defined by either of the methods.

**Conclusions:**

Although electrocardiographic criteria demonstrated poor diagnostic accuracy for LVH compared with TTE, both modalities were independently associated with adverse cardiovascular outcomes. These findings suggest that ECG-defined and TTE-defined LVH provide complementary prognostic information and may reflect distinct pathophysiological processes.

## What is already known on this topic

Multiple parameters, including ECG and cardiac imaging studies, can diagnose left ventricular hypertrophy (LVH). There is an important debate regarding the clinical significance of electrical versus anatomical hypertrophy, and doubt remains as to whether these are part of the same pathological process or represent different clinical phenomena.

## What this study adds

We demonstrate in this sample of Brazilian adults that there is little overlap between individuals with electrocardiographic and echocardiographic-defined LVH, and both are related to adverse cardiac outcomes even after mutual adjustment in multivariate prediction models.

## How this study might affect research, practice, or policy

Our study reinforces the notion that electrical hypertrophy may be a distinct clinical entity rather than simply part of the remodeling process of anatomical hypertrophy. These findings help clinicians understand various aspects of prognosticating patients with hypertension and show the independent capacity of the ECG to provide a prognosis even in the absence of anatomical hypertrophy.

## Introduction

Persistent hemodynamic overload is one of the main determinants of left ventricular hypertrophy (LVH). It may lead to anatomical and electrical maladaptations. While the first can be assessed by the transthoracic echocardiogram (TTE) that estimates left ventricle mass and geometry,^1^ the electrocardiogram (ECG) measures the latter by the registry of electrical activity. Despite being frequently considered equivalent, these may come from different adaptive mechanisms and may result in different manifestations and perhaps even different clinical consequences. Traditionally, LVH can be recognized in the 12-lead ECG by characteristic alterations in the QRS complex, ST segment, and T waves. Numerous ECG LVH diagnostic criteria have been developed based on these alterations. The detection of LVH by the ECG can be important for identifying target organ damage in hypertension and also for its prognostic value in predicting future cardiovascular disease (CVD).^2,3^ The different LVH ECG criteria have varying performance regarding their accuracy, specificity, and sensitivity, with most studies reporting low sensitivity and higher specificity when compared to the TTE.^4^ Although the low sensitivity poses a limitation for the value of ECG as a triage method for LVH in the general (or hypertensive) population, its specificity and possible prognostic information can be valuable in targeting individuals with a higher risk for CVD.^5^

Another aspect of ECG-detected LVH is its relationship to anatomical hypertrophy detected by imaging studies, such as the TTE. The concept of electrical versus anatomical hypertrophy has gained increasing attention and is a matter of significant debate, as it can inform us about different pathological processes.^5^ Thus, the ECG findings can potentially provide independent and perhaps additional information when compared to other cardiovascular propaedeutic modalities, reflecting other subjacent cellular anomalies that go beyond anatomical diagnosis of LVH. Since CVD is the primary cause of morbidity and mortality worldwide, this ubiquitous exam may play an essential role in healthcare and healthcare access. The Adult Longitudinal Health Study (ELSA-Brasil) is a prospective occupational cohort that aims to evaluate determinants of CVD and diabetes in the Brazilian population. The ECG and TTE were obtained at study baseline.

Our goal was to evaluate how LVH diagnosed by the ECG using the Minnesota code (MC) definition would perform regarding its diagnostic parameters [*i.e,* sensitivity, specificity, positive and negative predictive values (PPV and NPV), positive and negative likelihood ratios (LR+ and LR-)] when compared to the TTE in this population. Additionally, we aim to evaluate the prognostic value of each of these two tools and whether they can exert these predictive capabilities independently of each other.

## Methods

### Study design and participants

This study was designed with two complementary approaches: a prospective longitudinal arm and a cross-sectional diagnostic accuracy arm. The study sample was derived from the baseline of ELSA-Brasil, a multicenter cohort recruited between 2008 and 2010, comprising 15,105 active and retired employees from six public universities and research institutions located in the Northeast, South, and Southeast regions of Brazil, aged 35–74 years. The study design and baseline characteristics have been previously described.^6^ Participants were considered eligible if they had both an interpretable ECG and a TTE. The TTE was offered to all participants, and its acquisition and reading were performed prioritizing first older individuals (over 60 years of age) and also a random subsample of 10% of the remaining population. Individuals with cardiac pacemakers, Wolff-Parkinson-White (WPW) syndrome, or complete intraventricular conduction block (QRS >120 ms) on ECG were excluded. The flowchart of exclusions is represented in Figure 1*.* Among participants from the baseline visit of ELSA-Brasil who had undergone TTE and had available data for both echocardiographic and electrocardiographic LVH assessment, 2,926 were initially selected for analysis. Four individuals with a pacemaker, WPW, or complete intraventricular conduction block (QRS >120 ms) on ECG were excluded, leaving 2,922 cases. An additional 73 participants were excluded due to missing or uninterpretable ECG data (resulting from noise and artifacts). The final study sample comprised 2,849 participants.

**Figure 1:** – Exclusion flowchart.

### ECG acquisition

Trained and certified technicians acquired standard 12-lead ECGs. All participants were examined in a comfortable environment, with skin preparation when necessary, and electrodes positioned at standardized anatomical sites.^7^ Recordings were transmitted and stored at the ECG reading center of the *Universidade Federal de Minas Gerais* in Belo Horizonte/Brazil. A digital device (Atria 6100, Burdick, Cardiac Science Corporation, Bothell, WA, USA) was used, providing automated measurements of heart rate, duration, amplitude, and axes of P, QRS, and T waves, as well as QT and corrected QT intervals (QTc) and QT dispersion. ECGs were transmitted from the Atria 6100 to a dedicated server at the CL-ELSA/MG Reading Center, where they were archived for subsequent analysis using the Pyramis ECG management system (version 6.2.b, Cardiac Science Corporation, Bothell, WA, USA), allowing editing and interval measurements beat by beat. All ECGs underwent automated analysis for wave amplitudes and were coded according to the MC.^8^ Tracings classified as abnormal by MC were manually reviewed. Given the lack of unequivocal superiority of any single ECG criterion,^9^ we adopted MC-defined criteria, as all tracings were coded accordingly. LVH on ECG was defined by the presence of MC codes 3.1, 3.3, and their combination.

### TTE acquisition

Echocardiograms were performed in 19% of ELSA-Brasil participants. TTE was conducted in individuals aged >60 years and in a random 10% subsample of the entire cohort. All examinations followed standardized research protocols.^10^ Images were acquired using a Toshiba Aplio XG system with a 2.5-MHz sector transducer and transferred to the ELSA Picture Archiving and Communication System (PACS). Analyses were performed at the ELSA-Brasil echocardiography reading center. Readings included qualitative assessment of echocardiographic findings and quantitative measurements of left ventricular (LV) systolic and diastolic function, LV geometry and dimensions, left atrial size, regional wall motion abnormalities, and valvular disease. LV mass was calculated using the Devereux formula: $\mathrm{LVM}(g)=0.8\times [1.04\times (\mathrm{IVS}+\mathrm{LVIDd}+\mathrm{PWTd}{)}^{3}-(\text{ LVIDd }{)}^{3}]+0.6g$ , where IVS is interventricular septal thickness, LVIDd is LV internal diameter at diastole, and PWTd is posterior wall thickness at diastole. In this study, LVH was defined as LV mass index >115 g/m^2^ in men and >95 g/m^2^ in women.

### Predictive variables

For diagnostic accuracy assessment, predictive variables were categorized as LVH by ECG. LVH on TTE was represented by the variable LVH_TTE and was considered the gold standard for diagnosing LVH. We organized LVH on ECG by three variables, two of which represented a single MC criterion: LVH_ECG1 (Minnesota Code 3.1) and LVH_ECG2 (Minnesota Code 3.3), and the third, the sum of the two, LVH_ECG3 (combined 3.1 + 3.3).

Regarding the prognostic analysis, to investigate the potential incremental predictive value of combining ECG and TTE information, we created a composite variable LVH_ECG+TTE, defined as the sum of LVH_TTE and LVH_ECG3. In this analysis, all the other predictors were used: LVH_TTE, LVH_ECG1, LVH_ECG2, LVH_ECG3, and LVH_ECG+TTE.

### Covariables

No covariates were defined for diagnostic accuracy analyses. For the prognostic models, the following covariates were included: sex (male, female); educational attainment (with or without completed higher education); age group (35–44, 45–54, 55–64, 65–74 years); hypertension (systolic BP ≥140 mmHg and/or diastolic BP ≥90 mmHg, self-reported diagnosis, or use of antihypertensive medication); systolic blood pressure (SBP); diastolic blood pressure (DBP); diabetes mellitus (defined diagnosis, use of drugs for the treatment of diabetes, HbA1c >6.5%, fasting glucose >126 mg/dL, or postprandial glucose >200 mg/dL); dyslipidemia, defined in this analysis as any of the following: Total cholesterol >240 mg/dL, LDL >160 mg/dL, HDL <40 mg/dL, or use of lipid-lowering therapy; smoking (≥100 cigarettes lifetime and current smoking status); and self-reported CVD (heart failure, coronary heart disease, or stroke).

### Outcomes

For diagnostic accuracy analyses, outcome variables included sensitivity, specificity, PPV, NPV, LR+, and LR- compared to the detection of LVH by the TTE. For prognostic analyses, we utilized only fatal outcomes, which were the outcomes available at the time of our study (December 2013). They were categorized as fatal myocardial infarction (AMI), fatal stroke, and their sum (FMACE). Outcomes were adjudicated by ELSA-Brasil investigators according to internationally validated criteria. Follow-up was conducted through annual telephone interviews, supplemented when necessary by clinical visits or linkage to national health databases, including the Mortality Information System (SIM) and the Hospital Information System (SIH) of the Brazilian Ministry of Health.

### Statistical analysis

Sensitivity, specificity, PPV, NPV, LR+, and LR- were calculated for each criterion. For prognostic analyses, baseline associations between LVH on ECG and LVH on TTE were evaluated. Kaplan–Meier survival curves and the log-rank test were used to compare the cumulative incidence of the defined outcomes between participants with LVH defined by ECG and by TTE. A Cox proportional hazards model was applied to estimate hazard ratios (HRs) and 95% confidence intervals (CIs) for the association of LVH by ECG and TTE separately with the primary outcome. A hierarchical Cox regression model was then constructed, adjusting sequentially for sex and age (model 1), hypertension, diabetes, smoking, dyslipidemia (model 2), and finally, self-reported CVD (model 3). As an additional sensitivity analysis, Cox regression was performed with mutual adjustment: echocardiographic LVH was included as a covariate in models where ECG-LVH was the predictor, and vice versa (model 4). Sample characteristics were summarized using descriptive statistics. Clinical data were entered into a database using the Statistical Package for the Social Sciences (SPSS), version 22.0. Continuous variables were expressed as medians and interquartile ranges (Q1–Q3) and compared using the Mann-Whitney test. The categorical variables are described in terms of frequencies and compared using the chi-square test. A two-sided alpha <0.05 was considered statistically significant.

## Results

The study population consisted of a majority of women, with a median age of 62 years, and a high level of education. Approximately half of the participants had hypertension, and the majority had dyslipidemia. Table 1 summarizes clinical and demographic characteristics, and the main findings are summarized in the Central figure.

**Table 1:** – Clinical and sociodemographic characteristics of the study population

Characteristics	General population (2848)	TTE LVH	ECG1 LVH	ECG2 LVH	ECG3 LVH	TTE+ECG LVH
LVH (%)	-	12.5	4.1	1.9	5.9	17
Male participants (%)	46.3	41.7	58.6	66.0	60.9	46.9
Age group (%)						
35 - 44 years	7.1	1.1	6.9	17.0	10.1	4.1
45 - 54 years	16.0	4.2	6.9	3.8	5.9	5.0
55 - 64 years	43.1	44.0	41.4	39.6	40.8	44.0
65 - 74years	33.9	50.7	44.8	39.6	43.2	46.9
Age (median)	62 (56 – 66)	65 (61 – 69)	63 (60 – 67,8)	64 (59 – 69)	63 (60 – 68)	64 (61 - 68)
Scholarity						
Superior (%)	56	41,7	34,5	28,3	32,5	40,9
Hypertension (%)	49.8	77.0	72.4	62.3	69.2	73.5
Systolic arterial pressure (median)	123.5 (113 - 136)	135 (124 - 150)	135 (122 – 155)	135 (122 –153)	135 (122 - 154)	134 (122 - 150)
Diastolic arterial pressure (median)	75.5 (69 - 83)	78.5 (70 - 86)	79.3 (73- 88)	79.5 (71 - 88)	79.5 (72 - 88)	78.5 (71 - 87)
Smoking (%)	10.4	9.2	8.6	18.9	11.8	9.3
Diabetes (%)	26.2	43.3	37.1	24.5	33.1	40.4
Dyslipidemia (%)	56.0	60.4	53.4	62.3	56.2	58.6
Low HDL (%)	24.1	30.6	18.1	26.4	20.7	28.0
High LDL (%)	36.0	39.9	31.9	39.6	34.3	37.5
CVD (%)	11.2	27.8	12.9	13.5	13.1	23.4

TTE: transthoracic echocardiogram; LVH: left ventricular hypertrophy; ECG: electrocardiogram; PAS: pressão arterial sistólica; PAD: pressão arterial diastólica; HDL: high density lipoprotein; LDL: low density lipoprotein; CVD: cardiovascular diseases.

Of the 2,849 participants, 12.5% had LVH by echocardiography. LVH identified by the LVH_ECG3 definition (the sum of MC 3.1 + 3.3) was the most frequent, followed by LVH_ECG1 and LVH_2. These frequencies are described in Table 2. The composite variable LVH_ECG+TTE identified LVH in 17% of participants. Individuals with LVH by either modality were more likely to have hypertension and dyslipidemia. The performance of each LVH criterion is also summarized in Table 2. In general, they all yielded a poor sensitivity and higher specificity, as well as poorer PPV and better NPV.

**Table 2:** – Performance of the electrocardiogram for the diagnosis of left ventricular hypertrophy compared to the echocardiogram

	Prev.	S	E	PPV	NPV	LR+	LR -
ECG 1	4,1%	7%	96,3%	21,6	87,9	1,89	0,97
ECG 2	1,9%	5%	98,6%	32,1	87,8	3,57	0,96
ECG 3	5,9%	12%	94,9%	24,9	88,2	2,35	0,93

S: sensitivity; E: specificity; PPV: positive predictive value; NPV: Negative predictive value; LR +: positive likelihood ratio; LR -: negative likelihood ratio; ECG: electrocardiogram.

As demonstrated in Table 3, LVH_ECG1 was associated with the highest event rates for mortality due to AMI, stroke, and FMACE. Table 4 shows multivariate predictive models adjusted for sex, age, cardiovascular risk factors, and LVH. For AMI mortality alone, none of the variables remained statistically significant in the final model. However, both LVH_ECG1 and LVH_ECG3 were associated with a nearly threefold higher risk of stroke mortality. For the composite outcome of AMI and stroke mortality (FMACE), all variables except LVH_ECG2 were associated with increased risk. Kaplan–Meier survival curves for AMI, stroke, and combined fatal events are shown in Figures two, three, and four, respectively.

**Table 3:** – Outcome frequencies in the overall cohort and stratified by predictor variables

Mortality rate	All	LVH TTE	LVH ECG 1	LVH ECG 2	LVH ECG 3	LVH TTE + ECG
Death due to MI	0.7%	1.7%	1.7%	1.9%	1.8%	1.2%
Death due to stroke	0.9%	2.2%	3.4%	1.9%	3.0%	2.1%
Combined stroke and MI death	1.6%	3.9%	5.2%	3.8%	4.7%	3.3%

MI: myocardial infarction; LVH: left ventricular hypertrophy; TTE: transthoracic echocardiogram; ECG; eletrocardiogram.

**Table 4:** – Multivariate predictive models for the outcomes of death due to myocardial infarction, stroke, and combined

Hazard Ratio (HR) IC 95%	LVH TTE	LVH ECG 1	LVH ECG 2	LVH ECG 3	LVH TTE + ECG
HR MI					
Model 1 adjusted for sex and age	3.1 (1.2 – 8)	2.6 (0.6 – 11.4)	2.8 (0.4 – 20.9)	2.8 (0,8 – 9,6)	2.1 (0.8 – 5.5)
Model 2 = model 1 + CV risk factors*	2.9 (1.1 – 7.7)	2.1 (0.5 – 9.2)	2.1 (0.3 – 16)	2.2 (0,6 – 7,6)	1.9 (0.7 – 4.9)
Model 3 = model 2 + prevalent CVD	2.6 (0.9 – 7.3)	2.1 (0.5 – 9)	2.1 (0.3 – 15.7)	2.2 (0.6 – 7.5)	1.7 (0.6 – 4.6)
Model 4 = model 3 + LVH by ECG or TTE	2.5 (0.9 – 6.9)	1.9 (0.4 – 8.2)	1.7 (0.2 – 12.6)	1.9 (0.5 – 6.5)	
HR stroke					
Model 1 adjusted for sex and age	3.1 (1.3 – 7)	4.2 (1.4 – 12)	2.1 (0.3 – 15.3)	3.7 (1,4 – 9,7)	2.9 (1.3 – 6.4)
Model 2 = model 1 + CV risk factors*	2.7 (1.2 – 6.3)	3.6 (1.2 – 10.4)	1.7 (0.2 – 12.9)	3.1 (1,2 – 8,3)	2.6 (1.2 – 5.7)
Model 3 = model 2 + prevalent CVD	1.9 (0.8 – 4.6)	3.4 (1.2 – 9.9)	1.8 (0.2 – 13.1)	3 (1.2 – 8.1)	2 (0.9 – 4.4)
Model 4 = model 3 + LVH by ECG or TTE	1.8 (0.7 – 4.3)	3.3 (1.1 – 9.5)	1.5 (0.2 – 11.5)	2.8 (1.1 – 7.6)	
HR MI + stroke					
Model 1 adjusted for sex and age	3.2 (1.7 – 5.9)	3.6 (1.5 – 8.4)	2.4 (0.6 – 10.1)	3.4 (1,6 – 7,3)	2.7 (1.5 – 4.9)
Model 2 = model 1 + CV risk factors*	2.8 (1.5 – 5.4)	3 (1.3 – 7)	2 (0.5 – 8.1)	2.8 (1,3 – 6)	2.3 (1.3 – 4.3)
Model 3 = model 2 + prevalent CVD	2.3 (1.2 – 4.4)	2.9 (1.2 – 6.8)	1.9 (0.5 – 8.1)	2.7 (1.3 – 5.8)	1.9 (1.1 – 3.6)
Model 4 = model 3 + LVH by ECG or TTE	2.1 (1.1 – 4.1)	2.7 (1.1 – 6.4)	1.6 (0.4 – 6.7)	2.4 (1.1 – 5.3)	

MI: myocardial infarction; LVH: left ventricular hypertrophy; TTE: Transthoracic echocardiogram; ECG: Eletrocardiogram; CV: cardiovascular; CDV: cardiovascular diseases. *Risk factors for adjustment were hypertension, diabetes, smoking, and dyslipidemia.

**Figure 2:** – Kaplan Meier curves for time until death due to myocardial infarction.

**Figure 3:** – Kaplan Meier curves for time until death due to stroke or cerebrovascular accident (CVA).

**Figure 4:** – Kaplan Meier curves for time until death due to the sum of fatal myocardial infarction and stroke (FMACE).

In the sensitivity analyses, adjusting TTE_LVH models for ECG_LVH and vice versa, the predictive significance for stroke mortality of LVH_ECG1 and LVH_ECG3, as seen in model 3, remained significant. Similarly, in Model 4, variables previously associated with FMACE in Model 3 retained statistical significance.

## Discussion

Electrocardiographic criteria for LVH, when compared with TTE as the gold standard, demonstrated low diagnostic accuracy, mainly attributable to very low sensitivity. In addition, the likelihood ratios obtained were unfavorable, with positive values below five and negative values close to 1.0. Because likelihood ratios integrate both sensitivity and specificity, they are considered superior parameters for assessing a test’s ability to correctly influence clinical reasoning regarding the presence or absence of disease. The low concordance of ECG criteria for LVH observed in our study is consistent with previous evidence, including a systematic review of diagnostic accuracy studies on ECG criteria for LVH.^4^ Reported median sensitivities ranged from 10.5% for the Gubner index to 21% for the Sokolow-Lyon index, while median specificities ranged from 89% (Sokolow-Lyon) to 99% (Romhilt-Estes, five-point threshold). Levy et al.,^9^ in the Framingham Heart Study, evaluated 4,684 participants and reported an overall ECG sensitivity for LVH of 6.9% and a specificity of 98.8%. Similarly, in the MESA study, the assessment of 13 ECG-based LVH criteria revealed consistently low sensitivity (10–26%) and high specificity (88–99%).^11^

A diagnostic method with such low sensitivity (despite a high specificity) is unlikely to adequately discriminate between affected patients and healthy individuals, tending instead to classify the majority as healthy, thereby limiting its clinical usefulness as a diagnostic tool. Thus, despite being inexpensive, widely available, and free from direct adverse effects, the ECG is not an effective screening method for LVH. At least, when traditional LVH ECG criteria are used, and anatomical hypertrophy by TTE is taken as the reference standard.

Regarding prognostic implications, although ECG-defined LVH showed weak correlation with echocardiographic LVH, it remained significantly associated with fatal cardiovascular outcomes. Both TTE and ECG were robust predictors of death from myocardial infarction or stroke, even after adjustment for conventional cardiovascular risk factors and established CVD. Neither method retained statistical significance in the final model for myocardial infarction mortality, although a strong trend persisted for TTE-defined LVH (HR 2.6, 95% CI 0.9–7.3). These findings may be partly explained by the low number of these events and by the closer association of LVH with hypertension and, therefore, by the consequences more intimately related to hypertension, particularly stroke.

Sensitivity analyses reinforced the independent value of each modality: ECG-defined LVH remained a significant predictor even after additional adjustment for TTE-defined LVH, and vice versa. In addition, the combined variable (LVH by both ECG and TTE) did not show stronger associations with adverse outcomes than the individual modalities, suggesting complementarity rather than additive predictive value. Consistent with our findings, Sundström et al. demonstrated that ECG-defined LVH was a strong predictor of all-cause mortality, even after adjusting for echocardiographic LVH.^12^ Similarly, Leigh et al. reported that the coexistence of ECG- and TTE-defined LVH was associated with an increased risk of cardiovascular events.^13^ At the same time, Patel et al. found that both modalities independently predicted incident atrial fibrillation, regardless of established risk factors.^14^ Notably, the predictive capacity of ECG-defined LVH did not depend on the presence of echocardiographic LVH, supporting its role as an electrophysiological marker of cardiac abnormalities, independent of anatomical hypertrophy. Such low concordance between the two modalities while also maintaining similar prognostic information is a signal that, perhaps, both entities represent a real signal of cardiovascular abnormalities.

Taken together, these findings support the hypothesis that ECG and TTE may capture distinct aspects of cardiac pathology, which could be described as electrical overload (ECG-defined) versus anatomical adaptation to overload (TTE-defined). It is also worth mentioning that the TTE identifies the augmentation in LV mass and not the myocardial hypertrophy *per se*, which means that other processes of mass increase (such as what happens in amyloidosis) also lead to an increase in LV mass. Another potential explanation for this discrepancy is that ECG-defined LVH is mainly driven by voltage criteria (in our study), which can be influenced by external factors (such as thorax shape or other comorbidities). The presence of a similar prognostic signal to TTE-defined LVH points to a potentially intrinsic cardiac phenomenon. Previous studies have shown that QRS alterations in ECG-defined LVH represent a combination of structural and electrical remodeling.^15,16^ In contrast, echocardiographic LVH is almost entirely determined by increased LV mass, or even, occasionally, as a confounder, representation of increased mass not related to true hypertrophy, such as in amyloidosis. In this context, ECG should not be viewed merely as a less sensitive method for detecting LVH. If that were the case, no additional prognostic value would be expected when ECG-defined LVH is considered alongside echocardiographic LVH. Supporting this interpretation, our study showed little overlap between participants meeting ECG versus TTE criteria for LVH and no incremental predictive strength for fatal CVD when a combined variable was tested. Thus, despite potential differences in underlying pathology, both ECG- and TTE-defined LVH provided equally relevant prognostic information regarding stroke risk. This reinforces the clinical value of ECG, especially in settings with limited access to advanced cardiovascular diagnostics or cardiology specialists. Future prospective studies are warranted to clarify whether ECG-defined LVH reflects an early form of electrical remodeling preceding anatomical changes, or whether it represents a distinct pathological entity.

Our results should be interpreted in light of some limitations. First, only fatal outcomes were analyzed, resulting in a relatively low number of events and wide confidence intervals for the estimated effects. Second, we defined ECG-based LVH using the Minnesota Code, which, although widely applied in population-based studies for decades, is less relevant to routine clinical practice. Commonly used clinical criteria, such as Cornell, Sokolow-Lyon, or Romhilt-Estes, might have yielded results more directly applicable to practice. Nevertheless, the use of the Minnesota Code was pragmatic, given its consistent application in our cohort. Similarly, echocardiographic LVH was defined as indexed LV mass >115 g/m^2^ for men and >95 g/m^2^ for women; the dichotomous analysis is less sensitive than the continuous. Finally, the TTE sampling strategy (10% of a random subsample of the cohort plus the participants aged >60 years) may have introduced selection bias, and our cohort, which consisted of civil servants from universities and research institutions, may not be fully representative of the general Brazilian population. Strengths of our study include the use of hard and objective outcomes, thereby minimizing measurement bias, the standardization of ECG and TTE acquisition, and the application of multivariate models adjusted for potential confounders, improving the accuracy of effect estimates.

## Conclusion

ECG-defined LVH showed lower diagnostic accuracy to identify TTE LVH; yet, both modalities were independently associated with adverse cardiovascular outcomes, potentially in a complementary manner. Further research is needed to clarify the prognostic value of different LVH markers and to better understand the relationship—and possible overlap—between electrical and anatomical hypertrophy.
